# Intraspecific, ecotypic and home climate variation in photosynthetic traits of the widespread invasive grass Johnsongrass

**DOI:** 10.1093/aobpla/plaa015

**Published:** 2020-05-05

**Authors:** Shannen Kelly, Rebecca A Fletcher, Jacob N Barney

**Affiliations:** School of Plant and Environmental Sciences, Virginia Tech, Blacksburg, VA, USA

**Keywords:** Functional traits, invasive species, Johnsongrass, *Sorghum halepense*

## Abstract

Despite their near ubiquity across global ecosystems, the underlying mechanisms contributing to the success of invasive plants remain largely unknown. In particular, ecophysiological traits, which are fundamental to plants’ performance and response to their environment, are poorly understood with respect to geographic and climate space. We evaluated photosynthetic trait variation among populations, ecotypes and home climates (i.e. the climates from the locations they were collected) of the widespread and expanding invader Johnsongrass (*Sorghum halepense*). We found that populations vary in the maximum net photosynthetic flux and the light-saturated net photosynthetic rate, and that agricultural and non-agricultural ecotypes vary in apparent quantum yield and water-use efficiency (WUE). We also found that populations from warmer home climates had lower dark respiration rates, light compensation points and WUEs. As Johnsongrass expands across the USA the abiotic and biotic environments are driving variation in its genetics, phenotypes and its underlying physiology. Our study demonstrates the importance of evaluating physiological traits in invasive plants, especially as they relate to home climates.

## Teaser

Despite their near ubiquity across global ecosystems, the underlying mechanisms contributing to the success of invasive plants remain largely unknown. Here, we found that the widespread invasive grass Johnsongrass varies in maximum photosynthetic rates among diverse populations and ecotypes. We also found that populations from warmer home climates had lower dark respiration rates, light compensation points and water-use efficiencies. As Johnsongrass expands across the USA the abiotic and biotic environments are driving variation in its genetics, phenotypes and its underlying physiology. Our study demonstrates the importance of evaluating physiological traits in invasive plants, especially as they relate to home climates.

## Introduction

Invasive species cause broad ecological and economic damage worldwide (Vilà *et al*. 2011), and pose a growing threat with worsening global change (Simberloff 2000). Despite our broad understanding of invasive species, we still lack basic mechanistic information on what drives their success in most cases (Ren and Zhang 2009). Thus, there has been recent interest in identifying plant functional traits (e.g. leaf and growth traits) that facilitate invasion (Drenovsky *et al*. 2012), but less attention has been paid to the underlying physiology of invasive plants (McDowell 2002).

Photosynthesis is the basis for carbon accumulation, growth rates and reproduction, and readily responds both plastically and adaptively to biotic and abiotic factors. Photosynthetic traits have been shown to be higher in invasive than native species (McDowell 2002), and vary among populations along environmental gradients (Jonas and Geber 1999). For example, McDowell (2002) found that two invasive *Rubus* species had higher photosynthetic capacity and maintained higher net photosynthesis for longer than two co-occurring natives, though Feng *et al*. (2007) did not find differences in photosynthetic parameters between two invaders and one native across different irradiance levels. There is equivocal evidence on whether photosynthetic traits differ in invasive than native populations, as well as whether introduced populations differentiate among themselves post introduction, though it is possible that there exists intraspecific variation among invasive populations that contributes to their success in the introduced range.

Jonas and Geber (1999) showed intraspecific variation for physiological traits, including maximum photosynthetic rates. Brodersen *et al*. (2008) found high variability in photosynthetic rates among native and introduced genotypes of *Phalaris arundinacea*, suggesting physiological traits may be highly conserved. Arntz *et al*. (2000) found that herbicide-resistant biotypes had lower electron transport and water-use efficiency (WUE) than herbicide-susceptible biotypes, which translated into reduced photosynthetic rates of the resistant biotypes at >400 µmol photons m^−2^ s^−1^. Intraspecific variation in photosynthetic traits suggests there exists heritable variation among populations that may afford rapid adaptation to local conditions, assuming environmental variation has been removed as in a common garden (Arntz *et al*. 2000). However, there has yet to be a survey of intraspecific variation in photosynthetic parameters for a widespread invader with a large geographic distribution and relating those parameters to their diverse home climates and ecotype. Such a study may begin to shed light on the underlying mechanisms leading to range expansion and invasiveness (McDowell 2002).

*Sorghum halepense* (Johnsongrass) is a warm-season perennial C_4_ grass that currently occurs from 55°N to 45°S worldwide (Holm *et al*. 1977; Atwater *et al*. 2016). It has been described as one of the world’s worst weeds (Holm *et al*. 1977), including in the USA where it was one of the six most damaging agricultural weeds (McWhorter 1971), resulting in millions of dollars of lost revenue annually (McWhorter 1993). In the USA, Johnsongrass was likely introduced to the Southeast and Southwest independently (Sezen *et al*. 2016), subsequently spreading throughout the USA and becoming a dominant agricultural weed and roadside species (Atwater *et al*. 2016). Moreover, expanding Johnsongrass populations are differentiating genetically and phenotypically into unique agricultural and non-agricultural ecotypes (Atwater *et al*. 2016, 2018; Sezen *et al*. 2016). Atwater *et al*. (2016) found that agricultural populations grew larger than non-agricultural populations. They also found that performance varied by home climate—agricultural populations grew largest from cooler, wetter conditions. While some information exists on the relationship between climate and population performance (Atwater *et al*. 2016, 2018), no study has focused on the relationship between local climate variables and ecophysiological traits. As Meyerson *et al*. (2019) point out, an understanding of invasive species physiology at the individual level is critical to understanding current and future distributions and impacts.

Here, we aim to identify population and ecotypic variation in Johnsongrass ecophysiology across a large range of home climates in the introduced range of the USA. We predict higher photosynthetic rates in agricultural populations, as agricultural plants regularly grow larger than non-agricultural populations (Atwater *et al*. 2018). In agricultural systems, the ability to outcompete C_4_ crops may be intrinsically linked to Johnsongrass’ ability to accumulate biomass, and thus enhancements in the underlying photosynthetic capacity. We also expect photosynthetic parameters to vary by home climate, and thus populations, as others have found strong variation in phenotypes driven by home climate (Atwater *et al*. 2018), which likely has a physiological basis. By examining variation in Johnsongrass physiology of populations from across the USA, and relating this to climate parameters, we aim to determine if there is evidence of genetic differentiation of populations across habitat ecotypes (agricultural and non-agricultural). Evidence of local specialization may elucidate factors that contribute to the success of Johnsongrass across its vast and expanding range, as well as aid in the understanding of potential responses to climate change.

## Materials and Methods

Johnsongrass seeds from 14 populations spanning eight US states were chosen to represent a broad range of home climates and geography **[see****Supporting Information—Table S1****]**: Alabama (*n* = 1), Arizona (*n* = 2), Georgia (*n* = 1), Kansas (*n* = 1), Nebraska (*n* = 1), New Mexico (*n* = 2), New York (*n* = 1), Texas (*n* = 4) and Virginia (*n* = 1). Populations were also chosen to represent agricultural (i.e. sites used for active crop production; *n* = 8) and non-agricultural (i.e. roadside corridors and fields not used for agriculture; *n* = 6) ecotypes; as agricultural and non-agricultural habitats have been shown to vary in resource availability and frequency of disturbance (Kane and Rieseberg 2008), and we have increasing evidence of ecotypic differentiation in Johnsongrass (Atwater *et al*. 2016, 2018; Sezen *et al*. 2016).

To break physical dormancy, seeds were soaked in bleach for 4 h and rinsed with water for 1 h. Seeds were germinated in the laboratory in covered Petri dishes that contained small amounts of potting media on a heating plate set at 26.8 °C for 1 week. Following radical emergence, germinants were transplanted into 15 × 15 cm pots filled with Miracle Grow potting mix in a greenhouse on the Virginia Tech Campus, Blacksburg, VA. Greenhouse conditions were climate-controlled to ~30 °C and plants were watered twice daily using drip irrigation so that water would be non-limiting and with no supplemental lighting. Further, plants were randomized among benches. Each population had four replicates for a total of 56 individuals. Measurements were conducted on the plants once they had been in the greenhouse for a month so that leaf width was of adequate size to fill the chamber of the gas exchange system.

Plants were measured in a random order to reduce within-population variation caused by differences in time of day, daily temperature and humidity changes within the greenhouse that occur over the course of a day. Measurements occurred between 0800 and 1600 h in July and August 2018 in the greenhouse in which the plants were grown. Photosynthetic measurements were taken using the LI-COR 6400XT portable gas exchange system (LI-COR Inc., Lincoln, NE, USA) with a 6400-02B LED Light Source chamber and light-response curves were constructed following the LI-COR Light Response Curve Protocol. Prior to measurement, chamber conditions were set to maintain constant conditions throughout the measurement period: CO_2_ concentration was set to 400 ppm; block temperature to 25 °C; and humidity to under 50 % (**see****Supporting Information—Table S2** for details). Humidity, temperature and CO_2_ levels were chosen based upon standard values used in similar light-response curve studies and reviews (Long and Bernacchi 2003). Irradiance levels (μmol photons m^−2^ s^−1^) were set using a red/blue LED light source in the chamber as follows: 0, 50, 75, 125, 250, 500, 750, 1000, 1500, 2000, 2500, 3000 and 3250. For each replicate, the youngest, fully expanded leaf (~2.5 cm wide) was selected and dark-adjusted for 45 min before placing it in the chamber. After each leaf was placed in the chamber, it was allowed 5 min to stabilize before we initiated the measurements. The relationship between net photosynthetic rate and irradiance was used to construct photosynthetic light-response curves using a rectangular hyperbola Michaelis–Menten-based model using routines developed by Lobo *et al*. (2013). We extracted the following parameters from each fitted curve: maximum carbon assimilation rate (*A*_max_), apparent quantum yield (AQY), dark respiration rate (*R*_d_), light saturation point (LSP), stomatal conductance at the light saturation point (*g*_s_), light compensation point (LCPT), and light-saturated carbon assimilation rate (*A*_sat_) (Table 1; **see****Supporting Information—Figs S1****and****S2** for example photosynthetic light-response curves). We also estimated WUE for each replicate by dividing the photosynthetic rate by the transpiration rate measured at an irradiance of 2000 µmol photons m^−2^ s^−1^.

**Table 1.** List of parameters extracted and calculated from each light-response curve (Long and Bernacchi 2003).

Parameter	Definition	Description
*A*_max_	Maximum carbon assimilation rate	The asymptotic estimate of the maximum photosynthetic rate (µmol CO_2_ m^−2^ s^−1^)
AQY	Apparent quantum yield	Corresponds to the slope of the linear phase of the curve and indicates how efficiently solar energy is converted into chemical energy. Ratio of the number of photons emitted to the number of photons absorbed.
*R*_d_	Dark respiration rate	The flux of respiration when photon flux density = 0 (µmol CO_2_ m^−2^ s^−1^)
LSP	Light saturation point	The point at which O_2_ evolution levels off and there is no further increase in the flux of photosynthesis. Beyond this point, light is saturating for the photosynthetic process. The rate of CO_2_ uptake is no longer limited by photochemical but rather enzymatic processes and by the supply of CO_2_ (µmol photons m^−2^ s^−1^)
*g*_s_	Stomatal conductance	Stomatal conductance at the light saturation point (µmol m^−2^ s^−1^)
LCPT	Light compensation point	Irradiance at which photosynthetic CO_2_ uptake and respiratory CO_2_ release are at equilibrium (µmol photons m^−2^ s^−1^)
*A*_sat_	Light-saturated net photosynthetic rate	The net photosynthetic rate at which the light saturation point occurs (µmol CO_2_ m^−2^ s^−1^)
WUE	Water-use efficiency	Ratio of the number of units of carbon fixed to the number of units of water transpired (µmol µmol^−1^)

To investigate possible variation among populations and between ecotype, we conducted a nested ANOVA with the two fixed effects—population nested in ecotype and ecotype—for each of the extracted parameters. When the effect of population was significant, we used the R package ‘lsmeans’ to perform a Tukey test to conduct *post hoc*, pairwise comparisons between populations (Lenth 2016). Further, we assessed the effect of temperature and precipitation climate variables, ecotype and their interaction on the six extracted light curve parameters using ANCOVA. The following climate variables were chosen for their known impacts on Johnsongrass population variation (Atwater *et al*. 2016): total annual precipitation (MAP; mm); mean annual temperature (MAT; °C); and mean temperature of the warmest quarter (MTWQ; °C), the latter of which appears to be the most biologically relevant as it coincides with the growing season. Climate variables were estimated using historic (1955–2000) climate averages from the BIOCLIM database (Hijmans *et al*. 2005). We conducted separate ANCOVAs for each photosynthetic parameter. Additionally, to avoid model overfitting, we only assessed one climate variable at a time. All climate variables were transformed to be centred around the mean. Because population and climate are collinear, the effect of population was tested separately from climate. We use a *P*-value of ≤0.1 to indicate marginal significance. All analyses were conducted in R version 3.5.0 (R Core Team 2018).

## Results

**Figure 1.**
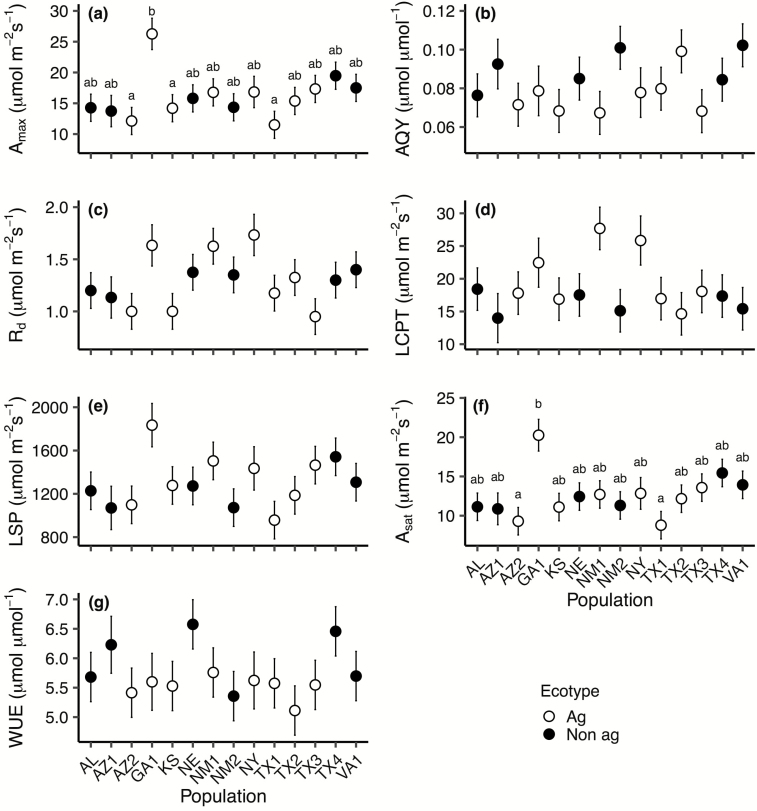
Least square means (± SE) of seven photosynthetic parameters extracted from photosynthesis light-response curves for the 14 Johnsongrass populations used in this study: (A) maximum photosynthetic rate (*A*_max_), (B) apparent quantum yield (AQY), (C) dark respiration rate (*R*_d_), (D) light compensation point (LCPT), (E) light saturation point (LSP), (F) net photosynthetic rate at the light saturation point (*A*_sat_) and (G) water-use efficiency (WUE). Lower case letters above the error bars indicate significant pairwise differences but only when the effect of population was significant (*P* ≤ 0.05). Open circles indicate the agricultural ecotype and closed circles indicate the non-agricultural ecotype.

There was a significant effect of population nested within ecotype for the photosynthetic parameters *A*_max_ (*P* = 0.016), *A*_sat_ (*P* = 0.020), *g*_s_ (*P* = 0.017) and to a lesser extent *R*_d_ (*P* = 0.055), indicating substantial variation among Johnsongrass populations for these parameters (Table 2; Fig. 1). The Georgia population consistently had higher parameter values than other populations, and likely drove population variance for *A*_max_ and *A*_sat_ (Fig. 1A and F). On the other hand, we did not find evidence of variation among populations for AQY, LCPT, LSP or WUE, but AQY, LCPT and WUE differed between ecotypes (Table 2).

**Table 2.** Results from a nested ANOVA (Model 1) and three ANCOVA (Models 2–4) models assessing the effects of population, ecotype, three climate variables (mean annual temperature, MAT, °C; total annual precipitation, MAP, mm; mean temperature of the warmest quarter, MTWQ, °C) and the interactions between ecotype and each of the climate variables on eight photosynthetic parameters extracted from photosynthetic light-response curves: maximum photosynthetic rate (*A*_max_), apparent quantum yield (AQY), dark respiration rate (*R*_d_), light compensation point (LCPT), light saturation point (LSP), net photosynthetic rate at the light saturation point (*A*_sat_) and water-use efficiency (WUE). Bold text indicates significant effects at *P ≤ 0.1 and **P ≤ 0.05.

		Model 1		Model 2			Model 3			Model 4		
		Ecotype	Eco./population	Ecotype	MAT	Eco. × MAT	Ecotype	MAP	Eco. × MAP	Ecotype	MTWQ	Eco. × MTWQ
	*df*	1	12	1	1	1	1	1	1	1	1	1
*A*_max_	*SS*	0.00	578.84	0.00	58.81	3.29	0.00	41.19	54.65	0.00	99.30	0.32
	*F*	0.00	2.48	0.00	2.26	0.13	0.00	1.63	2.16	0.00	3.93	0.01
	*P*	0.999	0.016**	0.999	0.139	0.724	0.999	0.208	0.148	0.999	0.053*	0.911
AQY	*SS*	0.002	0.01	0.003	0.000	0.000	0.003	0.000	0.002	0.003	0.000	0.000
	*F*	5.14	0.87	5.16	0.16	0.50	5.55	0.23	4.15	5.12	0.03	0.18
	*P*	0.029**	0.583	0.028**	0.691	0.485	0.023**	0.633	0.047**	0.028**	0.869	0.671
*R*_d_	*SS*	0.01	2.78	0.01	0.89	0.04	0.01	0.07	0.08	0.01	1.42	0.17
	*F*	0.04	1.97	0.04	6.75	0.30	0.04	0.45	0.53	0.04	12.00	1.47
	*P*	0.834	0.055*	0.843	0.012**	0.589	0.852	0.504	0.472	0.834	0.001**	0.232
LCPT	*SS*	147.48	632.92	147.48	196.15	57.03	147.48	1.08	66.66	147.48	218.65	69.69
	*F*	3.49	1.25	3.57	4.74	1.38	3.27	0.02	1.48	3.63	5.38	1.72
	*P*	0.069*	0.287	0.065*	0.034**	0.246	0.077*	0.878	0.230	0.063*	0.025**	0.196
LSP	*SS*	61819	2436721	61819	485099	130	61819	103227	20384	61819	590050	1567
	*F*	0.51	1.68	0.46	3.57	0.00	0.43	0.72	0.14	0.46	4.42	0.01
	*P*	0.478	0.109	0.503	0.065*	0.975	0.514	0.400	0.708	0.500	0.041**	0.914
*A*_sat_	*SS*	0.90	352.59	0.90	33.37	3.28	0.90	27.23	39.06	0.90	57.25	0.74
	*F*	0.07	2.39	0.06	2.06	0.20	0.06	1.74	2.50	0.06	3.63	0.05
	*P*	0.788	0.020**	0.815	0.158	0.655	0.812	0.193	0.120	0.812	0.063*	0.830
*g*_s_	*SS*	0.00	0.01	0.00	0.00	0.00	0.00	0.00	0.00	0.00	0.00	0.00
	*F*	2.15	2.46	1.61	1.49	0.83	1.79	1.93	6.01	1.65	3.36	0.38
	*P*	0.150	0.017**	0.211	0.228	0.368	0.188	0.171	0.018**	0.204	0.073*	0.541
WUE	*SS*	2.95	5.74	2.95	0.60	0.02	2.95	0.28	0.01	2.95	0.11	0.10
	*F*	4.20	0.68	4.45	0.90	0.03	4.40	0.42	0.01	4.39	0.16	0.14
	*P*	0.047**	0.758	0.040**	0.347	0.858	0.041**	0.521	0.928	0.041**	0.693	0.707

### Mean annual temperature

**Figure 2.**
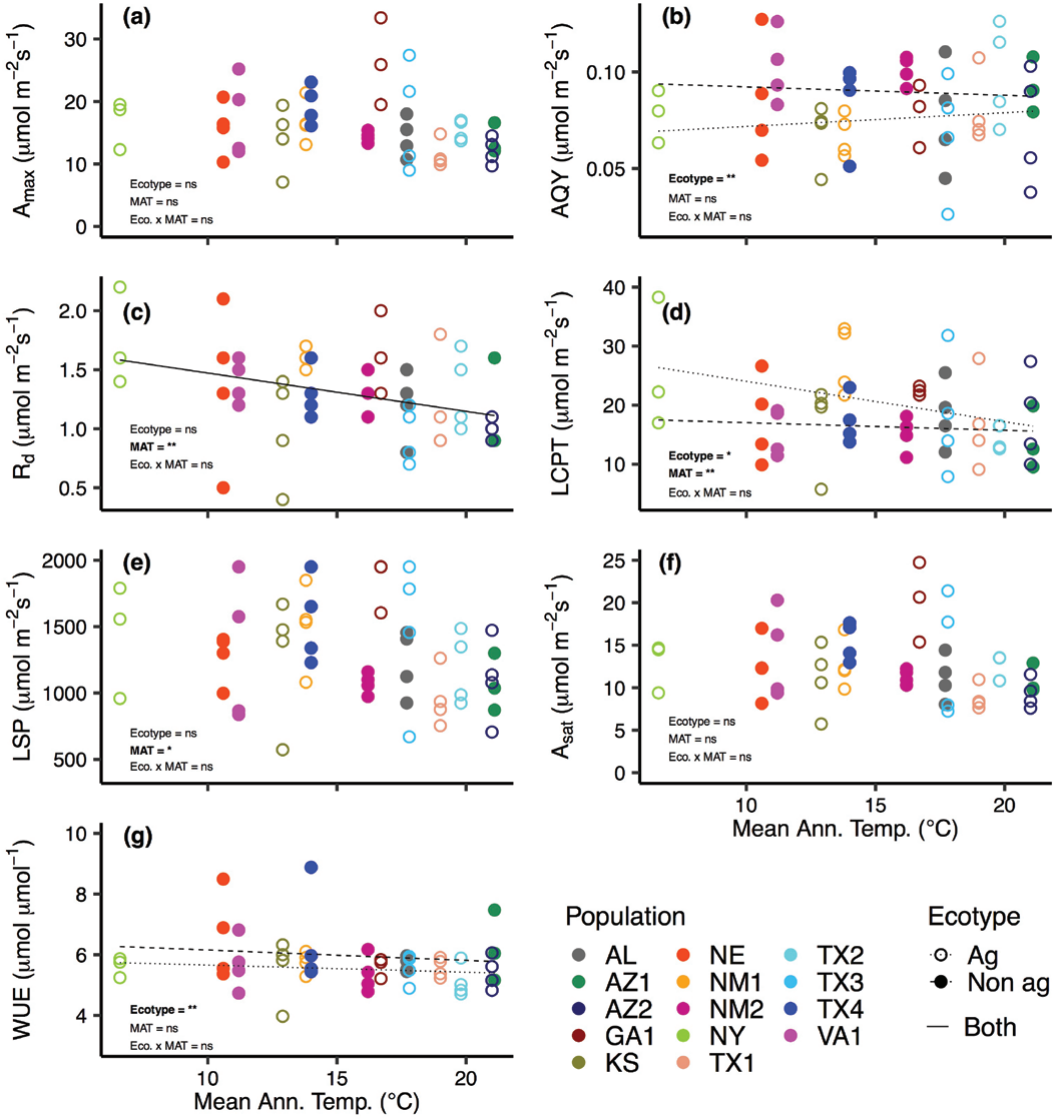
Relationship between mean annual temperature (MAT) and seven photosynthetic parameters extracted from the light-response curves of 14 Johnsongrass populations: (A) maximum photosynthetic rate (*A*_max_), (B) apparent quantum yield (AQY), (C) dark respiration rate (*R*_d_), (D) light compensation point (LCPT), (E) light saturation point (LSP), (F) net photosynthetic rate at the light saturation point (*A*_sat_) and (G) water-use efficiency (WUE). The agricultural ecotype is depicted with open circles and dotted lines and the non-agricultural ecotype is depicted with closed circles and dashed lines. No regression lines are shown if there was no significant relationship, two regression lines, one for each ecotype, are plotted if ecotype or the interaction between ecotype and MAT was significant, and a single regression line is shown if only MAT is significant. Bold text indicates the effect was significant (*P ≤ 0.10; **P ≤ 0.05).

**Figure 3.**
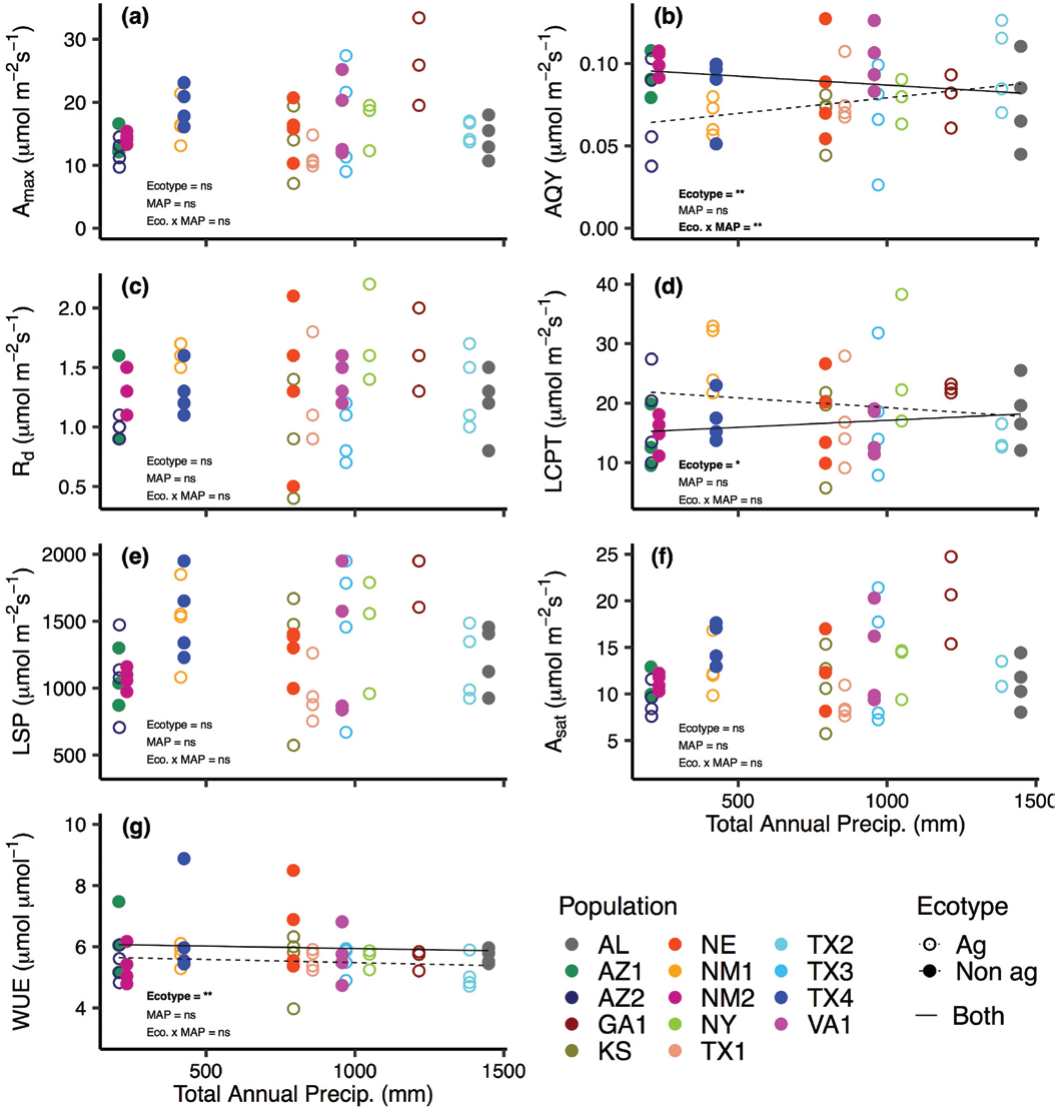
Relationship between total annual precipitation (MAP) and seven photosynthetic parameters extracted from the light-response curves of 14 Johnsongrass populations: (A) maximum photosynthetic rate (*A*_max_), (B) apparent quantum yield (AQY), (C) dark respiration rate (*R*_d_), (D) light compensation point (LCPT), (E) light saturation point (LSP), (F) net photosynthetic rate at the light saturation point (*A*_sat_) and (G) water-use efficiency (WUE). The agricultural ecotype is depicted with open circles and dotted lines and the non-agricultural ecotype is depicted with closed circles and solid lines. No regression lines are shown if there was no significant relationship, two regression lines, one for each ecotype, are plotted if ecotype or the interaction between ecotype and MAP was significant, and a single regression line is shown if only MAP is significant. Bold text indicates the effect was significant (*P ≤ 0.10; **P ≤ 0.05).

**Figure 4.**
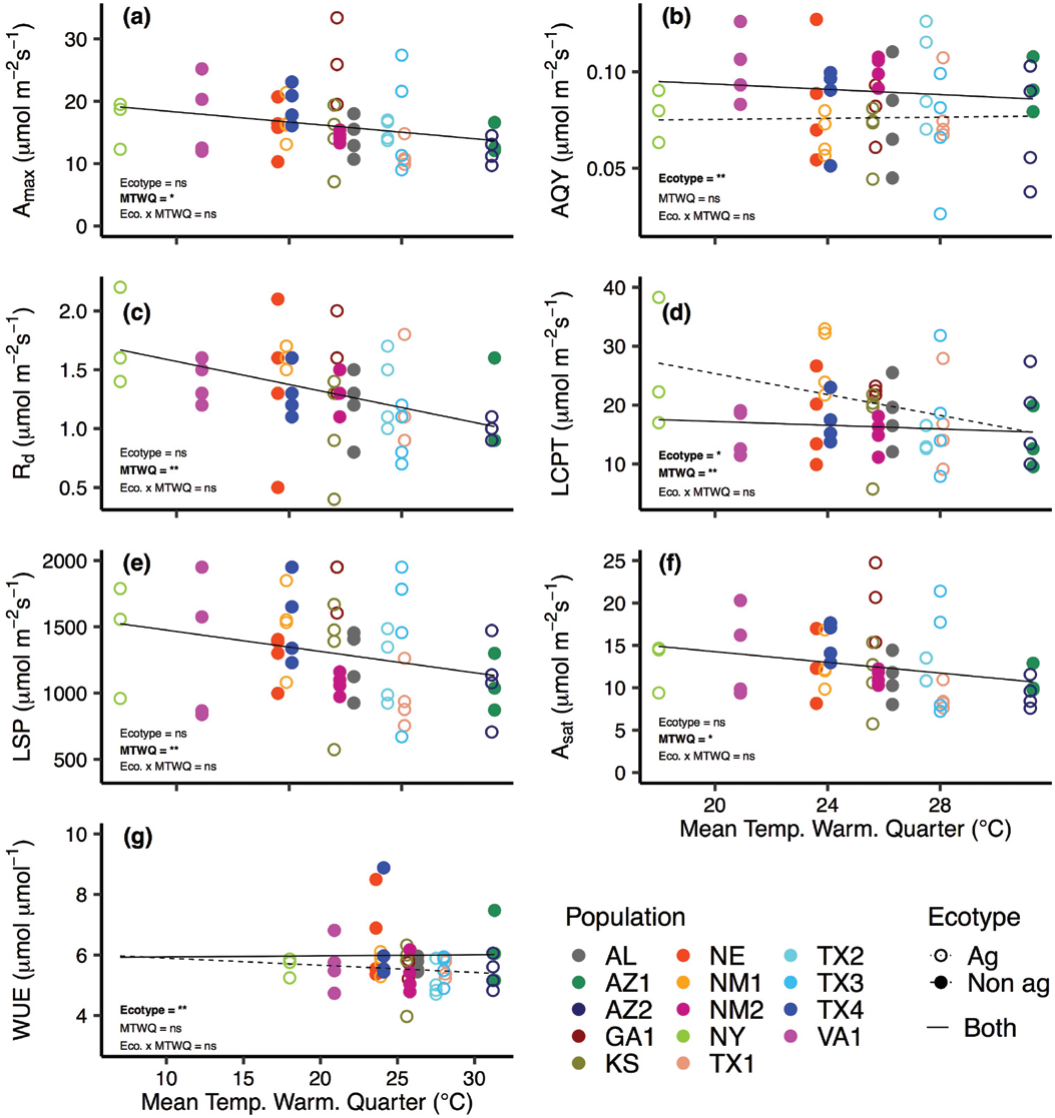
Relationship between mean temperature of the warmest quarter (MTWQ) and seven photosynthetic parameters extracted from the light-response curves of 14 Johnsongrass populations: (A) maximum photosynthetic rate (*A*_max_), (B) apparent quantum yield (AQY), (C) dark respiration rate (*R*_d_), (D) light compensation point (LCPT), (E) light saturation point (LSP), (F) net photosynthetic rate at the light saturation point (*A*_sat_) and (G) water-use efficiency (WUE). The agricultural ecotype is depicted with open circles and dotted lines and the non-agricultural ecotype is depicted with closed circles and solid lines. No regression lines are shown if there was no significant relationship, two regression lines, one for each ecotype, are plotted if ecotype or the interaction between ecotype and MTWQ was significant, and a single regression line is shown if only MTWQ is significant. Bold text indicates the effect was significant (*P ≤ 0.10; **P ≤ 0.05).

The LCPT (*P* = 0.034), LSP (*P* = 0.065) and *R*_d_ (*P* = 0.012) varied as a function of home site MAT (Table 2; Fig. 2), and we found significant differences between Johnsongrass ecotypes for AQY (*P* = 0.028), LCPT (*P* = 0.065) and WUE (*P* = 0.040) (Table 2; Fig. 2B, D and G). However, there were no significant interactions between Johnsongrass ecotype and MAT in relation to any of the physiological parameters. Dark respiration rate expressed an inverse relationship with MAT, indicating that the dark respiration rate of populations originating from cooler climates was higher than populations from warmer climates (Fig. 2C). Agricultural ecotypes exhibited AQY that was 11.7 % lower than non-agricultural ecotypes (0.08 ± 0.004 vs. 0.09 ± 0.005) when modelled against MAT (Fig. 2B), they also exhibited LCPT that was 20.4 % higher compared to non-agricultural ecotypes (20.0 ± 1.2 vs. 16.3 ± 1.4; Fig. 2D). Further, when modelled against MAT, non-agricultural populations demonstrated WUE values that were 8.7 % higher than agricultural populations (6.0 ± 0.2 vs. 5.5 ± 0.2; Fig. 2G), a trend that was similar for MAP (Fig. 3G) and MTWQ (Fig. 4G).

### Total annual precipitation

In the models assessing the effect of MAP and ecotype on the photosynthetic parameters, only AQY (*P* = 0.023), WUE (*P* = 0.041) and weakly, LCPT (*P* = 0.077) varied by ecotype (Table 2; Fig. 3). Additionally, there was a significant interaction between MAP and ecotype for AQY (*P* = 0.047) and *g*_s_ (*P* = 0.018; Table 2; Fig. 3B). Agricultural populations exhibited a positive AQY relationship with MAP while non-agricultural populations exhibited an inverse relationship with MAP, indicating that agricultural populations from drier climates expressed lower AQY values than non-agricultural populations from similarly dry climates (Fig. 3B). There was a positive relationship between stomatal conductance and MAP for agricultural populations and a negative relationship between stomatal conductance and MAP for non-agricultural populations.

### Mean temperature of the warmest quarter

We observed a significant inverse relationship between MTWQ and six photosynthetic parameters: *A*_max_ (*P* = 0.053), *R*_d_ (*P* = 0.001), LCPT (*P* = 0.025), LSP (*P* = 0.041), *g*_s_ (*P* = 0.073) and *A*_net_ (*P* = 0.063) (Fig. 4). Additionally, there was evidence of differences between ecotype for AQY (*P* = 0.028), LCPT (*P* = 0.063) and WUE (*P* = 0.041) (Table 2), indicating that, while non-agricultural ecotypes showed a 21.4 % lower LCPT than their agricultural counterparts, non-agricultural ecotypes exhibited AQY and WUE values that were 11.8 % and 8 % higher, respectively, than agricultural ecotypes (Fig. 4B, D and G).

## Discussion

It is increasingly recognized that our lack of understanding of the mechanisms contributing to successful invasion is an important research gap (Meyerson *et al*. 2019), in particular what role an invader’s underlying ecophysiology plays. Our study shows important population and ecotypic variation, some of which are strongly affected by the population’s home climate, in several physiological traits of the widespread invader Johnsongrass.

Through population genetic analyses Sezen *et al*. (2016) demonstrated that independent introductions of Johnsongrass occurred in the Southeastern and Southwestern USA. As Johnsongrass propagated throughout the USA, some sites of colonization, including initial sites, experienced recolonization. As a result, multiple introductions, followed by adaptation to local conditions and recolonization, may have increased genetic diversity outside of the initial founding populations. Complementing our previous work demonstrating strong population variation for various phenotypic traits, here we found Johnsongrass also varies in some physiological traits (Atwater *et al*. 2016, 2018; Sezen *et al*. 2016). For example, the photosynthetic parameters *A*_max_ and *A*_sat_ varied among Johnsongrass populations from across its introduced range of the USA (though largely drive by one population), though there was no trend relating these traits to their home climates.

We observed differences between agricultural and non-agricultural ecotypes for WUE, AQY and LCPT. Non-agricultural ecotypes consistently demonstrated higher WUE than their agricultural counterparts. Interestingly, the two ecotypes had inverse relationships between stomatal conductance, a key trait related to photosynthetic efficiency and WUE, and total home precipitation: agricultural populations had higher stomatal conductance with MAP, while non-agricultural populations had lower stomatal conductance with increasing MAP. As WUE is a mechanism that underlies the process of photosynthesis, significant differences between ecotypes may elucidate certain evolutionary adaptations that relate to water availability in their home habitats. Overall, agricultural environments are typically characterized as high-resource environments that are regularly subsidized with additional nutrients, irrigation in dry areas and reduced competition due to weed management. As such, it is plausible that weeds such as Johnsongrass, which originally spread throughout agricultural systems (Sezen *et al*. 2016), may have evolved as a luxury resource consumer (de Mazancourt and Schwartz 2012). The lack of selective pressure to be efficient with water would correspond to their lower WUEs. In contrast, non-agricultural systems, many of which are roadsides for Johnsongrass, experience a range of regular disturbances and are characterized by soils with low water-holding capacity, prompting non-agricultural ecotypes to adapt to fluctuating water availability through higher WUEs. Agricultural populations also had higher LCPTs, which typically characterizes plants found in high-light environments. Again, this likely represents the relatively low competition environments of agricultural fields.

We also found several instances of ecotypic variation as a function of home climate. For example, AQY values of non-agricultural ecotypes were significantly higher than agricultural ecotypes when modelled against home MAT and MTWQ. A significant interaction existed between ecotype and MAP; agricultural ecotypes from drier climates had lower AQY than non-agricultural ecotypes originating from similar climates. In conjunction, non-agricultural ecotypes exhibited significantly lower LCPT values than agricultural ecotypes, indicating that these populations transition from net negative to net positive carbon assimilation. Non-agricultural ecotypes start photosynthesizing with lower light exposure, reaching maximal photosynthesis quicker.

When framed in the context of biomass accumulation and WUE, these photosynthetic trends are logical. A previous study by Atwater *et al*. (2018) demonstrated that agricultural populations accumulated more dry biomass than Johnsongrass populations from non-agricultural populations. It is important to note that this study examined Johnsongrass at a mature (post-seedling, pre-reproductive) life stage. Johnsongrass has been shown to experience accelerated development at the seedling stage (Schwinning *et al*. 2017) and experience physiological changes during life stages that change the effectiveness of herbicide application (Enrique *et al*. 1999). A lower WUE in environments where water is not limited can translate into faster growth rates and more biomass accumulation. Under optimal, well-watered conditions, higher WUE often translates to lower stomatal conductance, lower assimilation rates and slower growth. On the other hand, plants with higher WUE will have higher stomatal conductance, higher assimilation rates and faster growth under optimal water conditions. Perhaps Johnsongrass performance varies depending on life stage and growing environment, and non-agricultural ecotypes are physiologically more efficient at this stage (post-seedling, pre-reproductive), which would support low LCPT values and high AQY values.

Furthermore, the high LCPT and low AQY of agricultural populations are related to its lower WUE. On a fundamental level, water use is a mechanism that controls fluxes of photosynthesis through carbon dioxide and water trade-offs. Similarly, slow rates of exchange would result in slower rates of photosynthetic flux. Additionally, at this life stage, there appears to be a trade-off in which agricultural populations are slow to accumulate biomass, but surpass non-agricultural ecotypes in biomass at the mature life stage (Atwater *et al*. 2018). Perhaps the pressure for establishment and persistence in non-agricultural fields results early high photosynthetic output for non-agricultural ecotypes, while agricultural ecotypes do not face this pressure at the seedling stage of development.

In addition, we observed a significant inverse relationship between *R*_d_ and MAT and *R*_d_ and MTWQ, in which populations from warmer climates exhibited lower dark respiration rates. Dark respiration is intrinsically linked to temperature, as many of the enzymes involved in the process are temperature-sensitive; in fact, heat-dependent reduction in Rubisco and reduced capacity for electron transport have been observed in relation to high temperatures (Hüve *et al*. 2011). Our measurements were all conducted at roughly the same temperature, precluding our ability to parse the role of temperature on enzyme kinetics.

Overall, many of these physiological traits varied among populations, ecotypes and home climates. Since we did not assess performance or fitness metrics in this study, it is difficult to make direct conclusions on how the intraspecific variation in physiological traits might translate into increased or decreased fitness. However, the physiological differences we observed here are consistent with growth and performance parameters from previous studies using agricultural and non-agricultural Johnsongrass ecotypes (Atwater *et al*. 2016, 2018). This suggests the trends we observed in the physiological traits likely translate into growth patterns observed under field conditions. Thus, we believe enhanced understanding of underlying ecophysiological traits will broaden our understanding of how invasive plants are capable of tolerating increasingly novel growing conditions as they expand their range. Clearly the large geographic, habitat and climate space that Johnsongrass has invaded in the USA have driven genetic, phenotypic and physiological variation. While exploring this variation in physiological traits enhances our understanding of Johnsongrass success, important gaps remain. In particular, it would be interesting to look at the intraspecific variation in alleles for the genes underlying these traits, as well as the plasticity of each trait. This in-depth exploration would inform not only our understanding for invasiveness, but also the potential for range expansion in the face of a changing climate.

## Supporting Information

The following additional information is available in the online version of this article—

**Table S1.** Geographic locations, ecotype (agricultural, non-agricultural) and home climate (mean annual temperature, MAT; total annual precipitation, MAP; mean temperature of the warmest quarter, MTWQ) of the 14 Johnsongrass populations used in this study.

**Table S2.** The range, mean and standard deviation (SD) of the CO_2_, temperature and humidity conditions in LI-COR 6400XT chamber during photosynthetic light-response curve measurements.

**Figure S1.** Example photosynthetic light-response curve for six non-agricultural Johnsongrass populations. Points represent values averaged (mean) across all non-agricultural populations.

**Figure S2.** Example photosynthetic light-response curve for the eight agricultural Johnsongrass populations used in this study. Points represent values averaged (mean) across all agricultural populations.

plaa015_suppl_Supplementary_Material

## Data

All data and associated code are available at the following citation: Fletcher, R., Barney, J. N., & Kelly, S. (2020). Data and Scripts for ‘Intraspecific, ecotypic, and home climate variation in photosynthetic traits of the widespread invasive grass Johnsongrass’ [Data set]. University Libraries, Virginia Tech. https://doi.org/10.7294/WPP2-9P95.
