# Supplementary material for: Intraspecific, ecotypic and home climate variation in photosynthetic traits of the widespread invasive grass Johnsongrass
Source: AoB Plants. 2020 May 5;12(3):plaa015. doi: 10.1093/aobpla/plaa015 (PMC7291798; doi:10.1093/aobpla/plaa015)
Supplement: plaa015_suppl_Supplementary_Material [file plaa015_suppl_supplementary_material.docx]

Table S1. Geographic locations, ecotype (agricultural, non-agricultural), and home climate (mean annual temperature, MAT; total annual precipitation, MAP; mean temperature of the warmest quarter, MTWQ) of the 14 Johnsongrass populations used in this study. Climate variables were derived from 30-year averages extracted from the BIOCLIM database with a spatial resolution of 1 km^2^ (Hijmans et al. 2005).

| Population | Ecotype | Latitude | Longitude | MAT (˚C) | MAP (mm) | MTWQ (˚C) |
| --- | --- | --- | --- | --- | --- | --- |
| Alabama | Non-Ag. | 32.225 | -88.158 | 17.7 | 1449 | 26.3 |
| Arizona 1 | Non-Ag. | 33.103 | -111.974 | 21.1 | 207 | 31.3 |
| Arizona 2 | Ag. | 33.000 | -112.087 | 21.0 | 209 | 31.2 |
| Georgia | Ag. | 33.525 | -83.444 | 16.7 | 1215 | 25.7 |
| Kansas | Ag. | 38.706 | -97.428 | 12.9 | 794 | 25.6 |
| Nebraska | Non-Ag. | 40.494 | -96.557 | 10.6 | 793 | 23.6 |
| New Mexico 1 | Ag. | 34.356 | -103.062 | 13.8 | 415 | 23.9 |
| New Mexico 2 | Non-Ag. | 32.202 | -106.732 | 16.2 | 234 | 25.8 |
| New York | Ag. | 42.764 | -75.552 | 6.6 | 1049 | 18.0 |
| Texas 1 | Ag. | 31.060 | -97.342 | 19.0 | 858 | 28.1 |
| Texas 2 | Ag. | 30.026 | -94.334 | 19.8 | 1385 | 27.5 |
| Texas 3 | Ag. | 33.280 | -96.893 | 17.8 | 970 | 28.0 |
| Texas 4 | Non-Ag. | 34.331 | -102.976 | 14.0 | 426 | 24.1 |
| Virginia | Non-Ag. | 37.194 | -80.574 | 11.2 | 957 | 20.9 |

Table S2. The range, mean, and standard deviation (sd) of the CO_2_, temperature, and humidity conditions in LI-COR 6400XT chamber during photosynthetic-light response curve measurements.

|  | Block Temperature (˚C) | Leaf Temperature (˚C) | CO_2_ Concentration (ppm) | Humidity (%) |
| --- | --- | --- | --- | --- |
| Range | 18.6-26.0 | 21.6-32.7 | 398.6-401.5 | 0.5-63.8 |
| Mean | 23.4 | 24.6 | 400.1 | 10.4 |
| sd | 1.5 | 1.4 | 0.5 | 9.7 |

Figure S1. Example photosynthetic-light response curve for six non-agricultural Johnsongrass populations. Points represent values averaged (mean) across all non-agricultural populations.

Figure S2. Example photosynthetic-light response curve for the eight agricultural Johnsongrass populations used in this study. Points represent values averaged (mean) across all agricultural populations.
